# Regulation of Human T-Lymphotropic Virus Type I Latency and Reactivation by HBZ and Rex

**DOI:** 10.1371/journal.ppat.1004040

**Published:** 2014-04-03

**Authors:** Subha Philip, Muhammad Atif Zahoor, Huijun Zhi, Yik-Khuan Ho, Chou-Zen Giam

**Affiliations:** Department of Microbiology and Immunology, Uniformed Services University of the Health Sciences, Bethesda, Maryland, United States of America; University of Pennsylvania School of Medicine, United States of America

## Abstract

Human T lymphotropic virus type I (HTLV-I) infection is largely latent in infected persons. How HTLV-1 establishes latency and reactivates is unclear. Here we show that most HTLV-1-infected HeLa cells become senescent. By contrast, when NF-κB activity is blocked, senescence is averted, and infected cells continue to divide and chronically produce viral proteins. A small population of infected NF-κB-normal HeLa cells expresses low but detectable levels of Tax and Rex, albeit not Gag or Env. In these “latently” infected cells, HTLV-1 LTR trans-activation by Tax persists, but NF-κB trans-activation is attenuated due to inhibition by HBZ, the HTLV-1 antisense protein. Furthermore, Gag-Pol mRNA localizes primarily in the nuclei of these cells. Importantly, HBZ was found to inhibit Rex-mediated export of intron-containing mRNAs. Over-expression of Rex or shRNA-mediated silencing of HBZ led to viral reactivation. Importantly, strong NF-κB inhibition also reactivates HTLV-1. Hence, during HTLV-1 infection, when Tax/Rex expression is robust and dominant over HBZ, productive infection ensues with expression of structural proteins and NF-κB hyper-activation, which induces senescence. When Tax/Rex expression is muted and HBZ is dominant, latent infection is established with expression of regulatory (Tax/Rex/HBZ) but not structural proteins. HBZ maintains viral latency by down-regulating Tax-induced NF-κB activation and senescence, and by inhibiting Rex-mediated expression of viral structural proteins.

## Author Summary

Most HTLV-1-infected individuals are asymptomatic. It is thought that the proviral DNA is transcriptionally inert and HTLV-1 replicates through mitotic expansion of host cells. The evolving provirus integration patterns in HTLV-1 carriers, however, suggest new infection occurs continuously. Whether or how HTLV-1 establishes latency and reactivates is unclear. We show that HTLV-1 infection in culture can lead to two alternative outcomes — productive infection accompanied by senescence or latent infection followed by clonal expansion — based on the relative expression of regulatory proteins: Tax, Rex, and HBZ. HTLV-1 latency is established by HBZ, and reactivation is achieved by Rex through regulating nuclear export of viral mRNAs. Elucidating mechanisms underlying HTLV-1 latency and reactivation can facilitate virus control to prevent progression to disease.

## Introduction

Human T-lymphotropic virus type 1 (HTLV-1) is a complex human retrovirus that infect approximately 10–20 million people worldwide [1]. In 3–5% of infected individuals a malignancy of CD4+ T cells known as adult T-cell leukemia/lymphoma (ATL) develops over a course of several decades [2], [3]. Other diseases caused by HTLV-1 include HTLV-1-associated myelopathy/tropical spastic paraparesis (HAM/TSP), HTLV-1 uveitis, and other inflammatory diseases.

Most HTLV-1-infected individuals become asymptomatic virus carriers. The prevailing view of HTLV-1 infection is that it is rather inactive and integrated HTLV-1 proviral DNA replicates largely through mitotic expansion of host cells. This view is based on three lines of evidence (see [4] for comments): (i) undetectable viral structural mRNA or protein expression in most infected PBMCs; (ii) undetectable cell-free viral particles in the plasma; and (iii) genetically stable viral genome due to very limited *de novo* infection through error-prone reverse transcription. However, longitudinal studies of HTLV-1 carriers indicate that the patterns of proviral DNA integration in PBMCs continue to evolve over time [5], suggesting that *de novo* infection of naïve cells occurs constantly in virus carriers (see [4] for a review). In infected individuals, there is also a robust CTL response against Tax and HBZ [6]–[8], implicating immune activation via persistent expression of viral antigens. Whether and how HTLV-1 establishes latency and reactivates is not understood.

HTLV-1 viral trans-activator Tax is a potent activator of viral mRNA transcription and the NF-κB pathway [3], [9]. We have shown previously that hyper-activation of NF-κB by Tax induces cellular senescence [10]. Remarkably, HBZ, a regulatory protein encoded by the HTLV-1 anti-sense transcript [11], dampens NF-κB activation [12] and thereby mitigates Tax-induced senescence [10]. These results raise the possibility that HTLV-1 infection may lead to two alternative outcomes dictated by the levels of Tax and HBZ [10]. When expressed at high levels, Tax drives robust viral replication, hyper-activates NF-κB, and triggers a senescence checkpoint response. Low levels of Tax and higher levels of HBZ, by contrast, result in moderation of NF-κB activation, prevention of senescence, and survival and persistence of HTLV-1-infected cells.

Our previous studies have shown that most HeLa and SupT1 cells infected by HTLV-1 in culture become senescent or arrested in cell cycle progression [13]. Here we demonstrate that HTLV-1 infection indeed can lead to productive infection with expression of all viral proteins, NF-κB activation, and senescence; or latent infection with expression of regulatory but not structural proteins. HTLV-1 latency is regulated by HBZ, which dampens LTR and NF-κB activation by Tax. The latter activity of HBZ prevents senescence induction and allows latently infected cells to proliferate. Interestingly, HBZ further inhibits Rex-mediated export of intron-containing viral mRNAs, thereby shutting off Gag, Gag-Pol, and Env expression, and virus production. The latent provirus can be reactivated by over-expressing Rex or down-regulating HBZ. Thus, the “latency state” of HTLV-1 in the cell culture system resembles that established by γ-herpesviruses such as EBV and KSHV, which express a handful of potentially oncogenic latency-associated viral proteins and RNAs that stimulate mitotic expansion of latently infected cells. We speculate that the persistent expression of Tax and HBZ during the early stage of HTLV-1 latency propels infected cells to proliferate.

## Results

### Alternative outcomes of HTLV-1 infection

**Figure 1:**
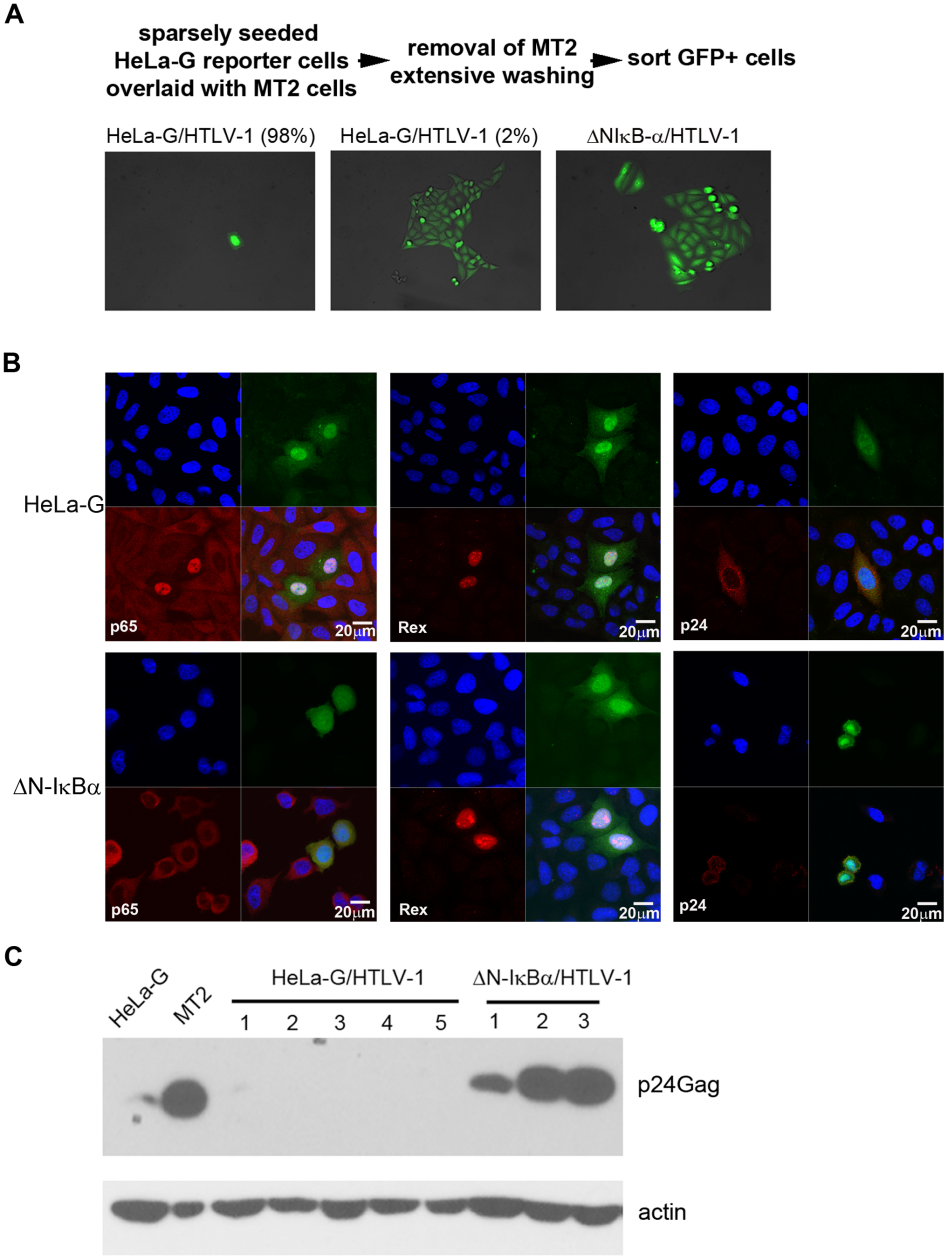
Isolation of HTLV-1 infected cell clones. (**A**) HeLa-G or HeLa-G/ΔN-IκBα (ΔN-IκBα) cells were co-cultured with mitotically inactivated MT2 cells for 48 hours as indicated by the flow diagram. Thereupon, GFP-positive cells were sorted and plated at low density such that proliferation of single cell could be monitored. Representative HTLV-1-infected HeLa-G and HeLa-G/ΔN-IκBα cells are shown. Most HTLV-1-infected HeLa-G cells (HeLa-G/HTLV-1) appeared as either single senescent cells (left panel, 98%), with a small fraction that continued to proliferate and formed colonies (middle panel, 2%). The majority of HeLa-G/ΔN-IκBα cells infected by HTLV-1 underwent expansion as GFP-positive colonies (right panel). It should be noted that HeLa-G/ΔN-IκBα cells are generally larger in size for reasons unclear at present. (**B**) P65/RelA localization, and Tax, Rex, and capsid protein p24 expression in HTLV-1-infected HeLa-G and HeLa-G/ΔN-IκBα cells. Cells were plated on chamber slides and infected with HTLV-1 by co-culturing with MT2 cells. After 48 hours, cells were fixed, permeabilized and immunostained for p65/RelA, Rex, and p24 (red). Nuclei were stained with DAPI (blue). HTLV-1-infected cells were identified by GFP expression (green). (**C**) Immunoblot analysis of expanded GFP-positive cell clones represented in middle and right panels of (A). Five HeLa-G/HTLV-1 clones and three HeLa-G/ΔN-IκBα/HTLV-1 were blotted for p24 capsid protein (p24) and β-actin (Actin) as shown.

We have previously derived a cell line known as HeLa-G that harbors a reporter cassette, 18×21-EGFP, consisting of the enhanced green fluorescence protein (EGFP) gene under the transcriptional control of 18 copies of the Tax-responsive 21-base-pair enhancer element [14]. HeLa-G cells express abundant GFP as a function of Tax expression either after HTLV-1 infection or transduction of the *tax* gene. We infected them by co-culture with mitotically inactivated HTLV-1-producing T-cell line, MT2 (Fig. 1A Top, see **Materials and Methods**), and found that most infected cells (98%) became senescent (Fig. 1A left panel). However, a careful examination of infected HeLa-G cells revealed that a small population (2%) continued to proliferate (Fig. 1A middle panel). Since our recent data indicate that hyper-activation of NF-κB by Tax is responsible for inducing cellular senescence [10], [15], we also tested the effect of HTLV-1 infection on a HeLa-G-derived cell line, HeLa-G/ΔN-IκBα in which the transcriptional activity of NF-κB is shut off by the stable expression of a degradation-resistant form of IκBα(ΔN-IκBα). As anticipated, HTLV-1-infected HeLa-G/ΔN-IκBα cells continued to proliferate after infection (Fig. 1A right panel). We also monitored infected HeLa-G and HeLa-G/ΔN-IκBα cells by immunofluorescence for p65/RelA, the capsid protein p24, and Rex at 48 hours after infection. As shown in Fig. 1B, NF-κB is activated in HTLV-1 infected HeLa-G cells as revealed by the localization of p65/RelA to the nucleus. Most of these cells became senescent as described above. By contrast, in HTLV-1 infected HeLa-G/ΔN-IκBα cells, p65/RelA is localized in the cytoplasm as a result of inhibition by ΔN-IκBα. Doublets of infected GFP-positive HeLa-G/ΔN-IκBα cells could be seen, indicative of cell proliferation. Both types of infected cells express Tax, Rex, and p24 as might be expected (Fig. 1B). Proliferating HeLa-G/HTLV-1 and HeLa-G/ΔN-IκBα/HTLV-1 clones were isolated by cell sorting based on GFP expression, expanded in cell culture, and characterized. Intriguingly, while the HeLa-G/ΔAN-IκBα/HTLV-1 clones expressed robust levels of p24 (Fig. 1B ΔN-IκBα/HTLV-1 clones 1–3), no p24 expression was detectable in HeLa-G/HTLV-1 clones (Fig. 1B HeLa-G/HTLV-1 clones 1 to 5). PCR analyses showed the chromosomal DNA of isolated clones to be positive for integrated HTLV-1 proviral DNA. Results from 3 (G1-3) and 2 (ΔN1-2) representative clones of each group are shown (supplementary Fig. S1). While all PCR products of ΔN1 and ΔN2 clones were of correct sizes as might be expected, G1-3 clones lacked (G1 and G2) or yielded a smaller *env* PCR product (G3) in the region that spans nucleotides 5318-5784 of the HTLV-1 genome (see supplementary Fig. S1 and Table S1), indicating gene deletions in this *env* region.

### NF-κB inhibition correlates with the proliferation of productively and latently infected cells

**Figure 2:**
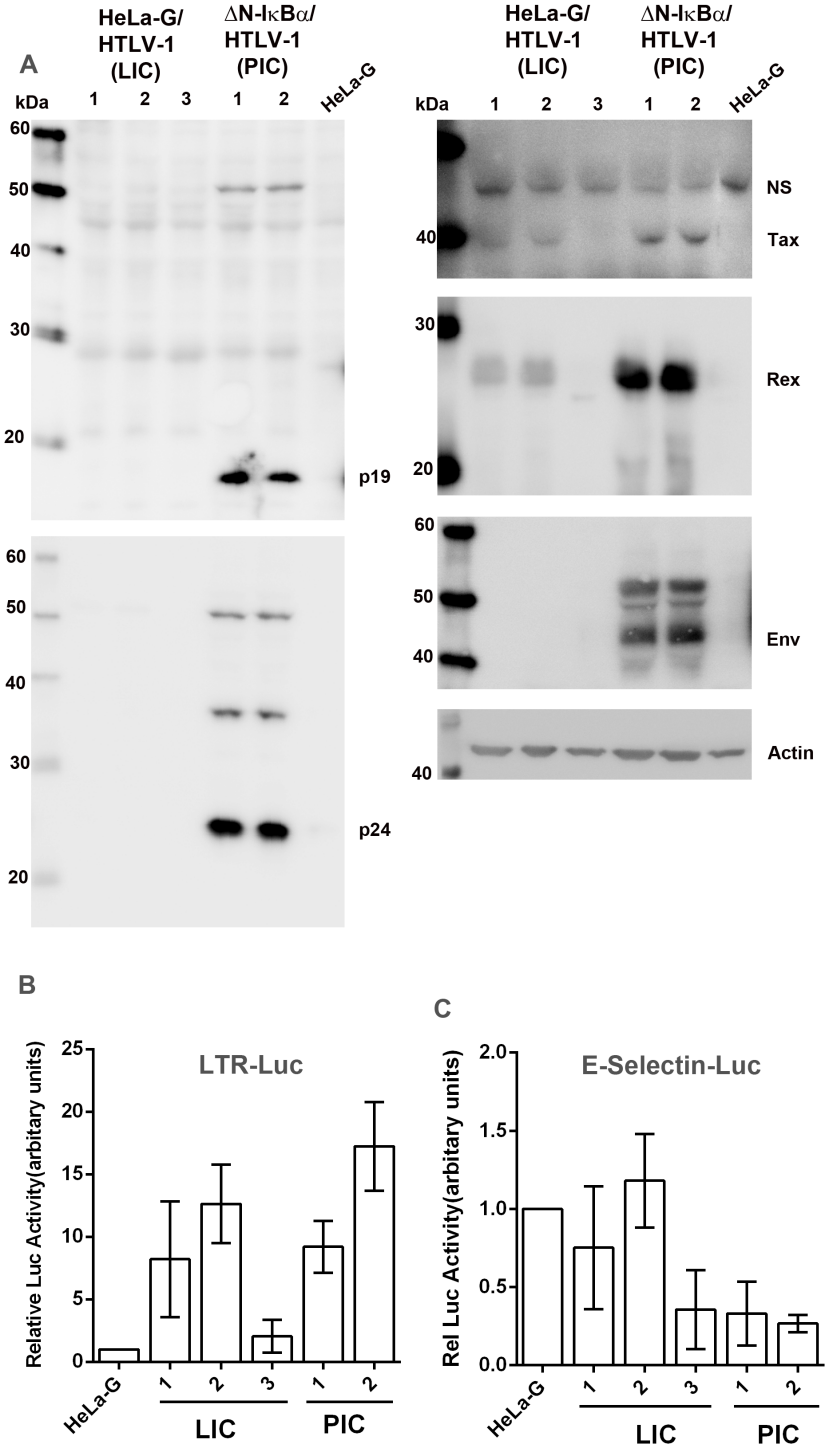
Characterizations of HeLa-G-derived cell lines productively (PIC, ΔN-IκBα/HTLV-1) or latently infected (LIC, HeLa-G/HTLV-1) by HTLV-1. (**A**) Immunoblot analyses of whole-cell lysates of HeLa-G/HTLV-1 clones 1, 2, and 3 (LIC); HeLa-G/ΔN-IκBα/HTLV-1 (ΔN-IκBα/HTLV-1, PIC) clones 1 and 2; and HeLa-G control. Antibodies used include p19, p24, Tax, Rex, Env, and β-actin (Actin). (**B & C**) LTR-Luc and E-selectin- Luc reporter activities in HTLV-1-infected cell lines analyzed in (**A**). Each reporter plasmid and the control Renilla-luciferase plasmid, pRL-TK, were transfected into HeLa-G cells, 3 LIC lines and 2 PIC lines as described in **Materials and Methods**. Relative luciferase activity after normalization is plotted.

As indicated by immunoblotting, most if not all HeLa-G/ΔN-IκBα/HTLV-1 cells were productively infected by HTLV-1 and abundantly expressed Gag (p24 and p19 matrix protein, abbreviated as p19), Env (gp46), Tax, and Rex (Fig. 2A). Together with the results described above (Fig. 1A left panel), these data indicate that cells productively infected by HTLV-1 usually undergo senescence as a result of chronic NF-κB activation by Tax. However, when NF-κB activity is blocked, the senescence response is prevented and the productively infected cell (PIC) population can grow and divide, and be established as individual virus-producing clones.

We next examined in depth the 3 cell lines derived from the minor population of proliferating HTLV-1-infected HeLa-G (HeLa-G/HTLV-1) cells whose NF-κB activity was unaltered. Interestingly, all three expressed Tax and Rex, albeit at lower levels (Fig. 2A right panels), but showed no detectable expression of p19, p24, or gp46 (Env) (Fig. 2A). The absence of Env from these clones correlated with *env* mutations detected by PCR (Fig. S1). Since only GFP+ cells were sorted and isolated, the positive detection of Tax expression is perhaps not surprising. These clones are designated as latently infected cells (LICs) for reasons that will become obvious later. It should be pointed out that Tax/Rex and HBZ expression is very low for LIC clone 3 (LIC3), where Tax expression is only detectable by the 18×21-EGFP reporter and Rex can only be seen by immunoblotting occasionally (see below).

**Figure 3:**
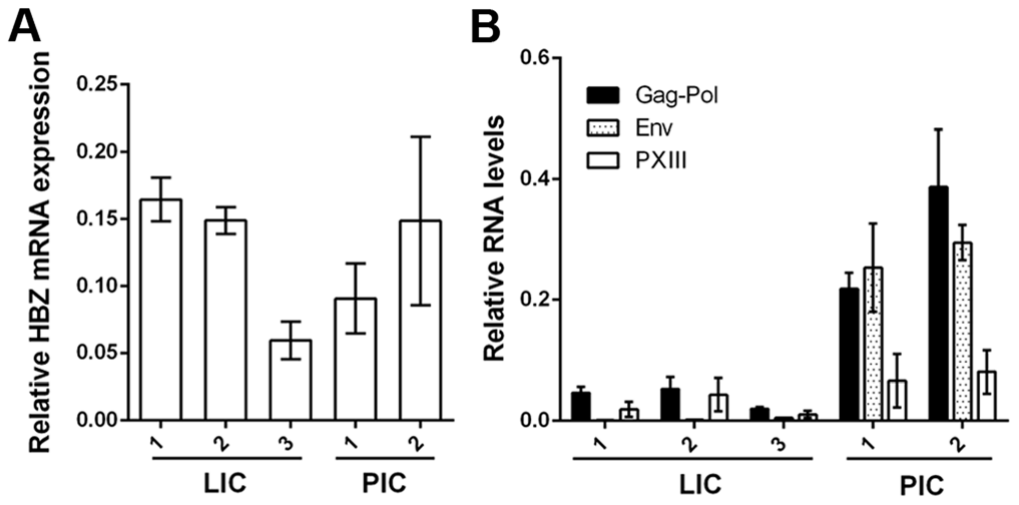
Expression of viral mRNA transcripts in LICs and PICs. Total RNA was prepared from each cell line, and cDNA was synthesized using HBZ antisense primer (**A**) or a cDNA synthesis supermix (**B**) followed by real-time PCR using gene specific primers for HBZ (**A**) and Gag-Pol, Env and pX regions (**B**). The results were normalized to β-actin mRNA in the same sample. Relative mRNA expression is represented. Primer sequences are listed in Table S2.

Activation of viral transcription by Tax is intact in LICs and PICs (albeit at a very low level for LIC3) as revealed by significant luciferase activities after transfection with an LTR-Luc reporter (Fig. 2B). This suggests that for LTR activation, the levels of Tax expressed in LICs and PICs are not limiting, with the exception of LIC3. This is as might be expected since each LTR has only 3 Tax-responsive 21-bp repeat elements (TxREs), and as the Tax/CREB complex recruited to the TxREs is known to have a high affinity for them, the effective concentrations of Tax necessary to drive LTR transcription need not be high. The lower levels of viral expression in LICs may be related to the chromosomal environments of proviral DNAs that dampen Tax-mediated trans-activation. As anticipated, the NF-κB activity in PICs is profoundly inhibited by the IκBα super-repressor (ΔN-IκBα) despite Tax expression. This is confirmed by the absence of detectable luciferase activity in them after transfection of an NF-κB reporter plasmid, E-selectin-Luc (Fig. 2C PIC lanes). Despite detectable Tax expression, NF-κB activity was not significantly induced in LICs (Fig. 2C). We think this is due to the expression of HBZ, which is known to down-modulate NF-κB activity, albeit not as drastically as ΔN-IκBα [10], [12]. Indeed, unspliced, but not spliced HBZ mRNA was readily detected and its levels are similar in both PICs and LICs (again, with the exception of LIC3) as determined by RT-PCR (Fig. 3A), consistent with the notion that its expression is independently regulated. The abundance of unspliced versus spliced HBZ mRNA most likely depends on the availability of splicing factors and can vary from cells to cells. Finally, it should be pointed out that although existing HBZ antibody can detect HBZ after DNA transfection, its sensitivity is insufficient for detecting HBZ during infection.

While the Tax/Rex mRNA (pXIII) levels in LICs were lower than those in PICs as indicated by mRNA quantitation (Fig. 3B, LIC1/PIC1 and LIC1/PIC2 about 1/3 and 1/4 respectively), greater differences were seen for Gag-Pol mRNAs (Fig. 3B, LIC1/PIC1 and LIC1/PIC2 about 1/5 and 1/8 respectively), Thus, more viral mRNAs of PICs are in the unspliced (Gag-Pol) form, and less so for LICs. The Env mRNAs in LICs were much lower (Fig. 3B). We think this is due to nonsense-mediated degradation caused by *env* mutations (Fig. S1). As mentioned earlier, the reduced Gag-Pol and Tax/Rex mRNA expression in LICs is most likely associated with the chromosomal sites of integration. The mRNA stabilization by higher levels of Rex in PICs [16], [17] likely also influences viral mRNA expression. Finally, inhibition of Rex-mediated nuclear export of intron-containing viral mRNAs in LICs contributes to the altered gene expression profile as elaborated below.

### Rex, and to a lesser extent, Tax, reactivates HTLV-1 from latency

**Figure 4:**
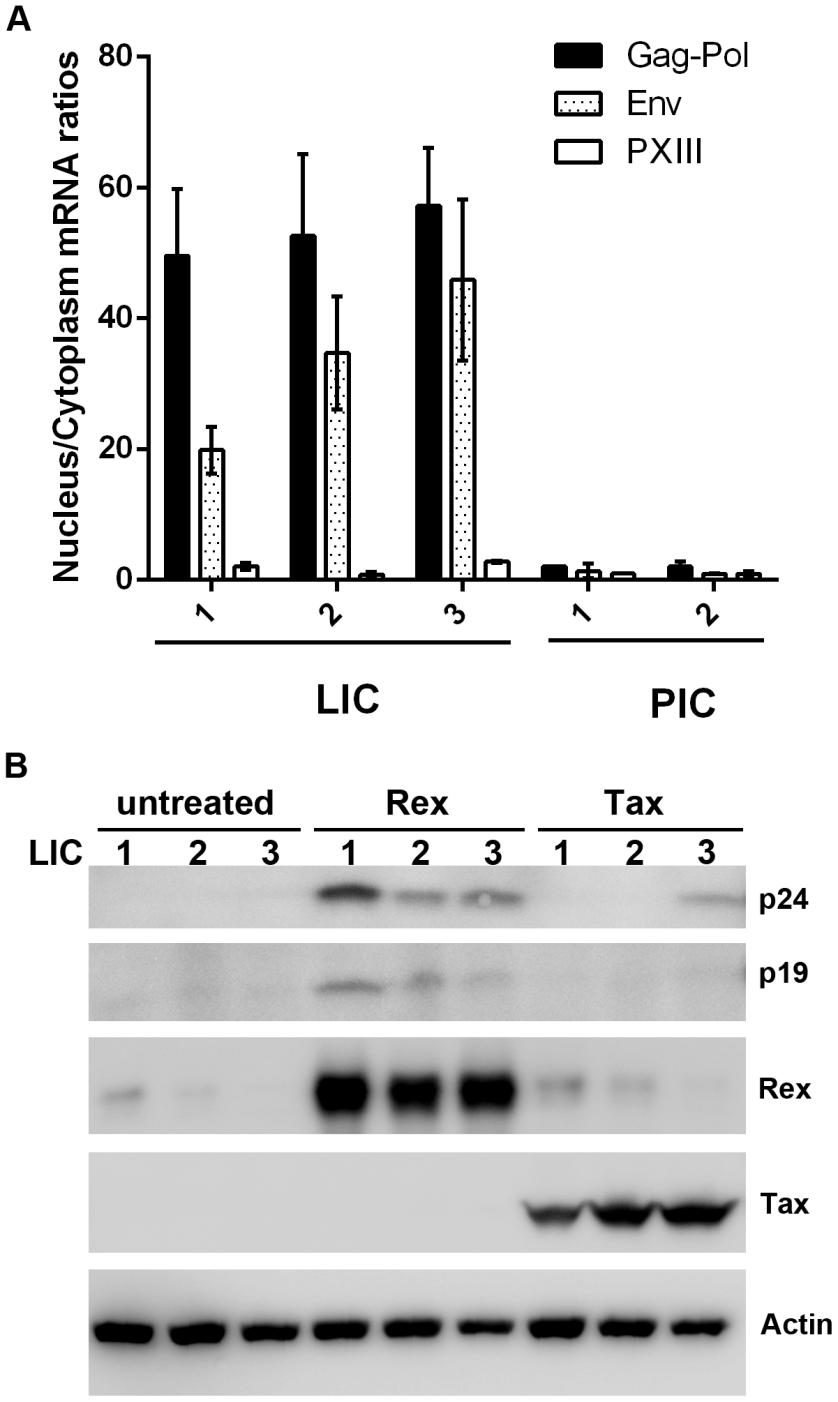
Nuclear export of intron-containing HTLV-1 mRNAs is blocked in latently infected cells, but can be rescued by Rex. (**A**) Nuclear to cytoplasmic ratios of Gag-Pol and pXIII mRNAs. The ratios were determined as in **Materials and Methods** by normalizing the level of the nuclear or cytoplasmic mRNA of interest to the respective nuclear or cytoplasmic β-actin mRNA in each mRNA preparation. (**B**) Reactivation of Gag expression in latently infected cell lines (LIC 1, 2, and 3) by Rex. Each of the three latently infected cell lines (3×10^5^ cells per well in a 6-well plate) were transfected with either a Tax (Bc12-Tax) or a Rex (pRSV-Rex) expression construct using Fugene reagent (Promega) for 48 hours. Whole cell lysates were analyzed by immunoblotting using antibodies against p24, p19, Tax, Rex, and β-actin (Actin).

Since the level of Rex is lower in LICs, we tested the possibility that their lack of Gag expression might be caused by a block in the nuclear export of unspliced viral mRNAs. Nuclear and cytoplasmic RNAs were fractionated for the LIC clones 1–3 and PIC clones 1 and 2, and subjected to qRT-PCR to quantify the nuclear and cytoplasmic levels of Gag-Pol, pX-III, and the control β-actin mRNAs. As indicated in Fig. 4A, there is a block in nuclear export of Gag-Pol mRNA in LICs with nuclear to cytoplasmic (N/C) ratios of approximately 30–40. By contrast, N/C ratios of Gag-Pol mRNA in PICs were approximately 1–2. The N/C ratios of the doubly spliced pX-III mRNA range from 0.6 to 2 in both cell types. Importantly, even though the levels of Rex in LICs were modest compared to those in PICs (Fig. 2A Rex panel on the right), it was detectable by immunoblotting, and was expected to export at least some unspliced Gag-Pol mRNAs. Intriguingly, Rex appeared altogether inactive in LICs.

We next asked if p24 expression could be reactivated by Tax or Rex in the LICs. Contrary to conventional wisdom, over-expression of Tax in LICs via transfection of an expression vector, Bc12-Tax, had very little impact on inducing p24 expression except in LIC clone 3 (Fig. 4B right 3 lanes). By contrast, when a Rex-expression plasmid was transfected, p24 expression was readily induced in all three LIC lines (Fig. 4B compare middle 3 and left 3 lanes). As expected, more robust reactivation of LIC3 could be achieved with co-transfection of both Rex and Tax (supplemental Fig. S2). These results again suggest that Tax is not a limiting factor for viral gene expression in many LICs. Importantly, they also suggest that the endogenous Rex in LICs may be defective or inhibited; and the defect or block can be complemented or overcome by over-expressing exogenous Rex.

### HBZ blocks Rex-mediated nuclear export of unspliced mRNAs

**Figure 5:**
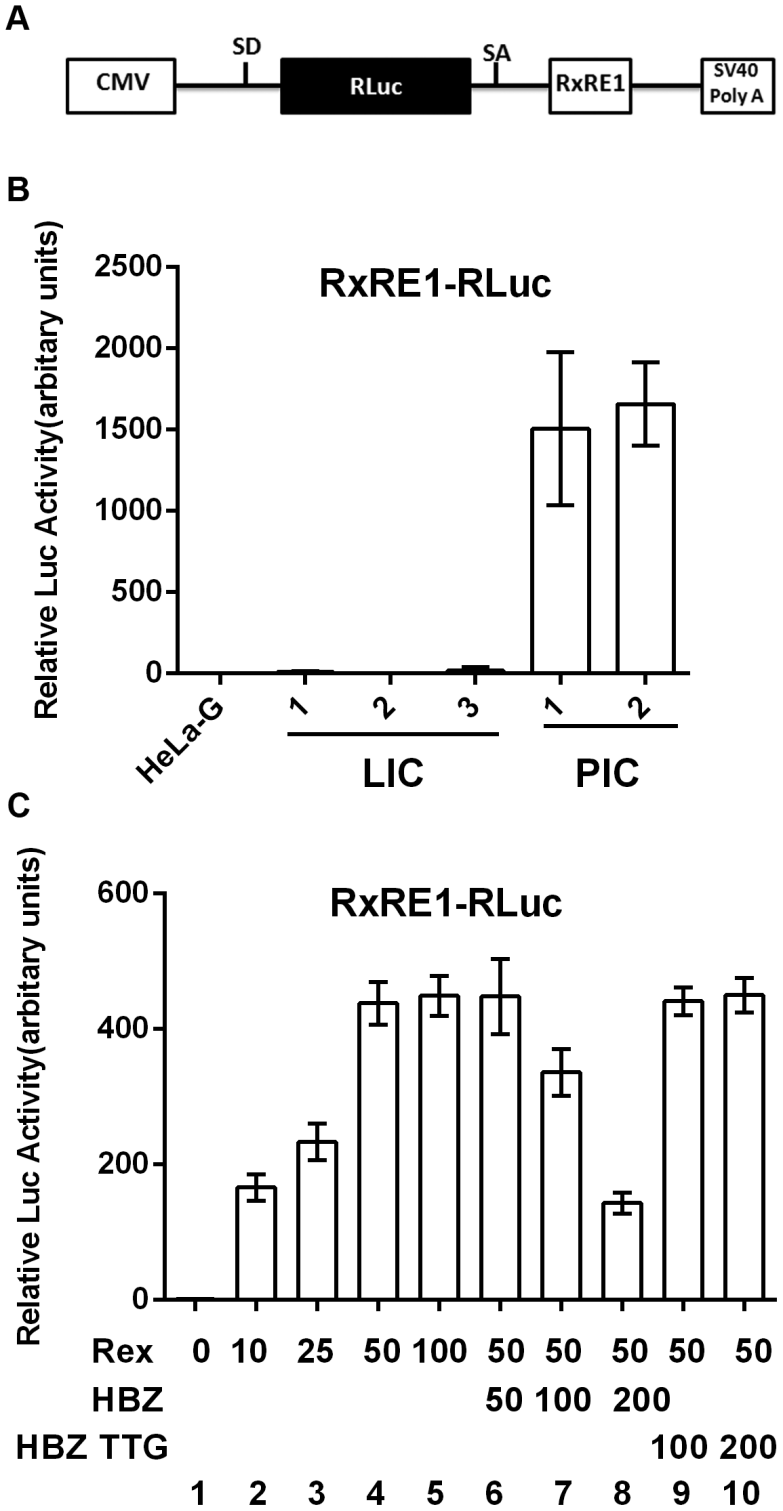
HBZ inhibits Rex-mediated nuclear export of intron-containing mRNAs. (**A**) Schematic representation of the Rex reporter construct, RxRE1-RLuc. CMV, cytomegalovirus immediate early enhancer/promoter; SD, splice donor; Rluc, Renilla luciferase; SA, splice acceptor; RxRE1, HTLV-1 Rex-response element; and SV40 poly A, polyadenylation site of the SV40 early gene. (**B**) Rex activities in HeLa-G, latently infected cell (LIC) clones 1–3, and productively infected cell (PIC) clones 1 and 2. Cells of each clone were transfected with 200 ng RxRE1-Rluc and 20 ng of PGL3-firefly-Luc (PGL3-Luc) for 48 hours. Dual luciferase assay was performed as in **Materials and Methods**. Relative luciferase activities were determined by calculating Rluc/Luc ratios followed by normalizing against that ratio of HeLa-G control. Mean luciferase activity ±standard deviation from three experiments is plotted. (**C**) Dose-dependent inhibition of Rex activity by HBZ. HeLa-G cells were co-transfected with 200 ng RxRE1-Rluc and 20 ng PGL3-Luc together with the indicated amount of Rex and HBZ expression constructs. Dual luciferase assay was performed as in (B). Lanes 1–5, dose-dependent titration of Rex. Lanes 6–8, dose-dependent inhibition of the Rex activity by wild-type HBZ. Lanes 9 and 10 contained the HBZ TTG mutant whose translational initiation codon ATG is mutated to TTG.

To determine if the activity of Rex was blocked or defective in LICs, we transfected both LICs and PICs with an HTLV-1 Rex reporter plasmid, pRxRE1-RLuc (Fig. 5A upper panel; [18]). This reporter encodes an mRNA that contains the HTLV-1 Rex-response element (RxRE1) in the 3′ end, and an intron that harbors the coding sequence for the Renilla luciferase (RLuc). In the absence of Rex, the RLuc sequence is removed by splicing. The mRNA exported to the cytoplasm is therefore without Rluc, hence no luciferase activity is expressed. In the presence of Rex, however, the RxRE1-RLuc-intron-containing mRNA is exported to the cytoplasm and translated to yield Renilla luciferase. Indeed, pRxRE1-RLuc-transfected PICs readily produced Renilla luciferase activity (Fig. 5B PIC 1 and 2), consistent with their chronic production of viral structural proteins facilitated by higher levels of Rex. By contrast, RxR1E-RLuc-transfected LICs expressed little luciferase activity (Fig. 5B LIC 1–3), in agreement with the notion that Rex is either defective or inhibited in LICs. No luciferase activity is detectable in transfected control HeLa-G cells (Fig. 5B leftmost lane).

We next asked if the lack of Rex activity in LICs might be due to inhibition by a trans-acting viral factor. Of all viral proteins, we thought HBZ to be the most likely to have a role in inhibiting the nuclear export function of Rex. This is because low or no NF-κB activation was detected in LICs despite Tax expression, suggesting that HBZ was expressed in LICs and was inhibiting NF-κB activation and senescence induction as previously proposed [10], [12]. Since HBZ is already playing a critical role in rendering possible the continuous proliferation of Tax-expressing cells [10], it is logical that it might additionally prevent virus production so as to establish latency.

To test if HBZ could block nuclear export of unspliced mRNA by Rex, we titrated Rex and pRxRE1-RLuc plasmids to determine the minimal amount of Rex DNA needed to achieve maximal reporter activity (Fig. 5C lanes 1–5). That amount of Rex (50 ng) was then used in co-transfection with increasing amounts of an HBZ-expressing plasmid. Indeed, a dose-dependent reduction in Renilla luciferase activity was observed when Rex and pRxRE1-RLuc reporter were co-transfected with HBZ (Fig. 5C lanes 6–8), suggesting that HBZ blocked Rex-mediated export of RexRE1-RLuc-intron mRNA. This effect of HBZ is mediated by the HBZ protein and not mRNA, because an HBZ mutant with the ATG translational start codon mutated to TTG failed to block the activity of Rex (Fig. 5C lanes 9 and 10).

### Down regulation of HBZ and inhibition of NF-κB reactivate latent HTLV-1

**Figure 6:**
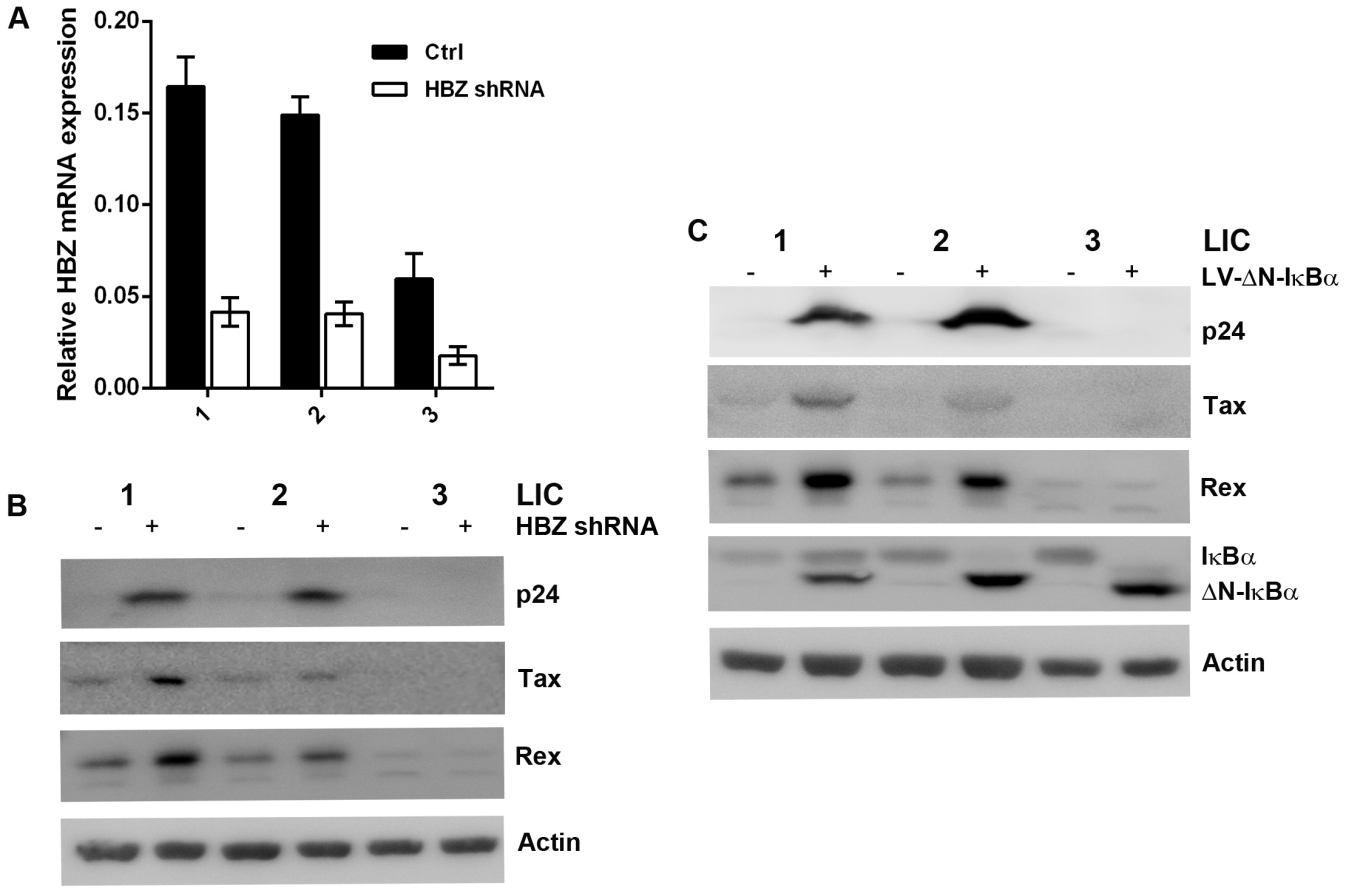
Knockdown of HBZ expression or strong inhibition of NF-κB reactivates latent HTLV-1. (**A**) Knockdown of HBZ mRNA expression in latently infected cells (LICs). LIC clones were transduced with a lentiviral HBZ shRNA construct (open bars) or with a control vector (solid bars) as previously described [30]. Transduced cells were selected in liquid medium containing 1 µg/ml puromycin. Knockdown of HBZ expression was confirmed by real-time PCR as detailed in **Materials and Methods**. (**B**) Induction of p24 expression in latently infected cell (LIC) clones after HBZ knockdown. LIC clones 1–3 and their derivatives stably transduced with lentiviral HBZ shRNA were analyzed by immunoblotting for p24, Tax, Rex, and β-actin (Actin). (**C**) Strong inhibition of NF-κB reactivates HTLV-1 from latently infected cells. LIC clones were transduced with a lentiviral construct, LV-ΔN-IκBα, that expresses a degradation resistant super-repressor form of IκBα, ΔN-IκBα. Transduced cells were selected as in (A) and analyzed by immunoblotting for the indicated proteins.

To confirm that HBZ is indeed responsible for preventing Gag expression in LICs, we derived a puromycin-selectable lentiviral vector encoding an HBZ-targeting shRNA under the transcriptional control of the Pol III-dependent snRNA U6 promoter. LICs were transduced with the LV-HBZ-shRNA-SV-puro (Fig. 6A & B, HBZ shRNA) or the empty vector, and selected in puromycin-containing medium for seven days. Down-regulation of HBZ mRNA in the HBZ-shRNA-treated cells was confirmed by qRT-PCR (Fig. 6A). As expected, after HBZ knockdown, an increase in Tax/Rex expression was observed (Fig. 6B), consistent with the notion that HBZ down-regulates viral gene expression [19]. Importantly, p24 expression was significantly induced and readily detected for LIC clones 1 and 2, indicating viral reactivation (Fig. 6B). LIC clone 3 did not show appreciable p24 expression, most likely because its level of sense mRNA transcription was too low. These results demonstrate that HBZ is responsible for down-regulating Tax-mediated viral sense mRNA transcription, and blocking Rex-mediated nuclear export of intron-containing HTLV-1 mRNAs and expression of viral structural proteins in LICs. It is important to note that in order for HBZ to exert effective control over viral replication, low levels of Tax/Rex expression are needed.

The levels of Rex and Tax in PICs are 3- to 4-fold higher than those in LICs (Fig. 2A right panels). While one cause for this difference may be the sites of proviral integration, since the major difference between LICs and PICs is the profound NF-κB inhibition by ΔN-IκBα in the latter, we thought strong NF-κB repression might contribute to the increased expression of Rex and Tax, and thereby reactivated latent HTLV-1 genome. Indeed, stable expression of the IκBα super-repressor, ΔN-IκBα, in LIC clones up-regulated Rex and Tax expression, and reactivated the latent proviruses as indicated by the induction of p24 expression, especially for LIC clones 1 and 2 (Fig. 6C). The mechanism by which NF-κB inhibition induces Rex and Tax expression is currently under investigation.

## Discussion

**Figure 7:**
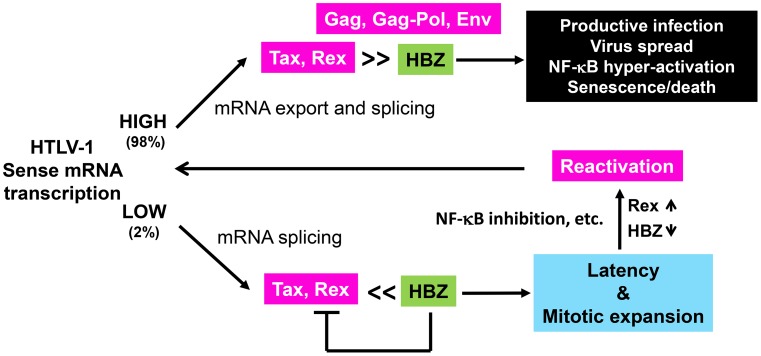
Alternative outcomes of HTLV-1 infection. Most HTLV-1-infected cells express Tax and Rex strongly. Under this condition, HBZ is insufficient to block the activities of Tax and Rex. As such, HTLV-1 structural proteins are abundantly expressed and viral particles are produced. In these cells, Tax chronically activates NF-κB, which triggers a cellular senescence response. A small fraction of HTLV-1-infected cells harbor latent proviral DNA. The levels of Tax/Rex expression are low in these cells. LTR trans-activation and senescence induction by Tax, along with viral mRNA nuclear export by Rex are inhibited by HBZ. As such, no viral structural proteins are expressed and these latently infected cells undergo mitotic expansion possibly driven by Tax and HBZ. Latent HTLV-1 provirus can be reactivated by down-regulation of HBZ, up-regulation of Rex, and strong inhibition of NF-κB.

In this paper, we present evidence to demonstrate that HTLV-1 infection can lead to two alternative outcomes based on the relative expression of Tax/Rex and HBZ (summarized in Fig. 7). In most HTLV-1 infected cultured cells, Tax/Rex expression is robust and viral structural proteins are abundantly expressed. In this condition, IKK/NF-κB is hyper-activated by Tax, triggering a host senescence response. When senescence induction is prevented by inhibiting NF-κB, cell clones productively infected by HTLV-1 can be readily established (Fig. 1). On the relatively rare occasions when Tax/Rex expression is weak, HBZ moderates NF-κB activation by Tax [10], [12], thus averting the host senescence response and allowing infected cells to continue to proliferate. Importantly, HBZ additionally inhibits Rex-mediated nuclear export of intron-containing mRNAs, thereby shutting off Gag, Gag-Pol, and Env production and committing infected cells into latency (Fig. 7). In latently infected cells, viral regulatory proteins, Tax, Rex, and HBZ, but not structural proteins are persistently expressed. Reactivation of Gag, Gag-Pol, and Env expression is achieved through up-regulation of Rex or down-regulation of HBZ (Fig. 7). Most interestingly, strong inhibition of NF-κB increases Rex and Tax expression and reactivates HTLV-1 replication.

We did not investigate the involvement of other HTLV-1 accessory proteins including p21Rex, p12I, p13II, and p30II in the present model. P30II is a nuclear and nucleolar protein thought to be a post-transcriptional modulator of viral replication [20]. Published data suggest that p30II retains the doubly-spliced Tax/Rex mRNA in the nucleus and thereby down-modulates viral gene expression by reducing the levels of Tax and Rex [20]. It has also been shown to interact with CBP/p300 and interfere with LTR trans-activation by Tax [21]. The continuous expression of Tax and Rex in LICs, albeit at low levels, indicates that the Tax/Rex mRNA is not sequestered. Importantly, the LICs described here were identified by virtue of Tax-driven GFP expression via the 18×21-EGFP reporter cassette. Cells that had no Tax/Rex expression would not have been scored in this system. Thus, a study of the latency state where Tax/Rex expression is completely silenced by p30II requires other approaches.

An unexpected finding from the present study is the high frequency with which LIC clones were found to harbor *env* mutations (Fig. S1). Additional attempts to isolate LIC clones that contain fully functional proviral DNA were not successful. This contrasts with the PIC clones, most if not all of which readily express Tax/Rex, Gag and Env. Whether the profound NF-κB inhibition in HeLa-G/ΔN-IκBα cells is responsible for shutting off innate host defense mechanism(s) that target mutations to retroviral genomes remains to be investigated.

The outcomes of HTLV-1 infection reported herein can adequately explain data from clinical and *in vivo* studies [4], [22]. In this model, cells productively infected by HTLV-1 immediately enter into senescence (Fig. 7; and ref. [10]) and most likely become eliminated by cytotoxic T lymphocytes [22]–[24]. Removal of senescent cells by NK cells is also a likely possibility [25], [26]. The latently infected cells, however, continue to express Tax, Rex and HBZ and can rely on the mitogenic activities of Tax, and HBZ protein and/or mRNA to drive cell proliferation. This is in accordance with the detection of Tax/Rex mRNA in a small population of infected cells reported previously [6], and the robust CTL response against Tax and HBZ seen in infected individuals [6], [8], [27]. The dynamically evolving proviral integration patterns in asymptomatic HTLV-1 carriers can now be explained by viral reactivation and *de novo* infection of naïve cells brought about via up-regulation of Rex and/or down-regulation of HBZ expression. In this model, it is necessary for latently infected cells to express only muted levels of Tax/Rex such that their activities can be controlled by HBZ. Indeed, proviral DNA integration sites in asymptomatic carriers were found mostly to locate in transcriptionally silent regions of chromosomes [28]. Finally, the observation that strong NF-κB inhibition can increase Rex expression and viral reactivation has clinical implications. Bortezomib, a proteasome inhibitor that inhibits NF-κB by stabilizing IκBα, has been entered into clinical phaseI/II trials for ATLL. Although ATLL cells in general no longer replicate HTLV-1, latently infected cells most likely persist in patients and may be reactivated by NF-κB inhibitors so as to influence the course of the disease. Based on present data, administration of antivirals to prevent HTLV-1 reactivation and spread may be advisable when bortezomib is used for ATLL treatment.

Given the alternative outcomes of HTLV-1 infection, do ATL cells emerge from productively or latently infected T lymphocytes? We think productive HTLV-1 infection of T lymphocytes whose senescence checkpoint response has been impaired is most likely the first step in ATL development. Such precancerous lymphocytes can express sufficient levels of Tax to overcome HBZ inhibition and achieve persistent IKK/NF-κB activation without inducing senescence. Through the loss of senescence response, the proliferative and survival advantages conferred by Tax-driven NF-κB activation, and the mitogenic activity of HBZ, such lymphocytes can readily undergo clonal expansion. This view agrees with recent high-throughput DNA sequencing data showing that most proviral integrations in ATL cells occur in transcriptionally active regions in the sense orientation [28]. As Tax is a primary CTL target, the loss of Tax and forward (sense) viral gene expression from precancerous T lymphocytes is selected and occurs through 5′ LTR DNA methylation, nonsense mutations, and deletions [29]. However, after Tax and Tax-dependent NF-κB activation are lost from pre-cancerous T lymphocytes, the impairment to the senescence checkpoint response remains, and can facilitate the evolution of Tax-independent NF-κB activation. As the expression of HBZ mRNA and protein is independently regulated, they can continue to exert mitogenic effect to propel the proliferation of cancerous cells [30]–[32]. Understanding how Rex, Tax, and HBZ expression is altered by cell signaling and cellular physiology to affect latency establishment and viral reactivation can facilitate the control of viral infection to prevent progression to disease.

## Materials and Methods

### Generation of HTLV-1 infected clones

HTLV-1 infections were performed in a 10 cm dish by co-culturing HeLa-G or HeLa-G/ΔN-IκBα cells (1–2×10^6^) with HTLV-1-producing MT2 cells (3×10^6^) that have been mitotically inactivated by mitomycin C (MMC) treatment (10 µg/ml for 2 hours). The co-culture was carried out in the presence of polybrene (8 µg/ml) for 16 hours. MT2 cells were then removed by washing with phosphate buffered saline (PBS). Fresh media was added and cells were grown for an additional 24 hours, and then harvested. GFP-positive cells were isolated using a cell sorter (BD FACSAria) housed in a lamella flow hood under aerosol-protection condition. Sorted cells were plated at low density on a 15-cm dish. Individual proliferating colonies were picked after a week into 96-well plates and further screened for the integrated HTLV-1 genome by PCR.

### Immunofluorescence

HeLa-G or HeLa-G/ΔN-IκBα cells grown on chamber glass slides were infected with HTLV-1 as described above. Forty eight hours after infection, cells were fixed with 4% paraformaldehyde, permeabilized with 0.1% Triton X-100, and immunostained overnight with the indicated primary antibodies followed by Alexa Fluor 568 secondary antibodies (Invitrogen, Carlsbad, CA.) Nuclei were counterstained using 4′,6′-diamidino-2-phenylindole (DAPI). The slides were then mounted in a fluorescence mounting medium (Dako). Images were captured using a Zeiss Pascal inverted confocal microscope.

### Immunoblotting

Standard procedures were used for immunoblotting. Typically, 30–50 µg of total proteins was used in each sample. The HTLV-1 Tax hybridoma monoclonal antibody 4C5 had been described previously [15]. The rabbit polyclonal antibody against Rex was a generous gift of Dr. Gisela Heidecker. HTLV-1 p24 antibody was purchased from Advanced Bioscience, HTLV-1 p19 and HTLV-1-gp46 (Env) antibodies were from Zeptometrix, IκBα, β-actin, goat anti-mouse, and goat anti-rabbit HRP conjugated secondary antibodies were from Santa Cruz.

### Genomic DNA isolation and PCR-analysis

HTLV-1-infected cells generated as above were harvested, washed, and dissolved in lysis buffer (50 mM Tris, pH 8.0, 10 mM EDTA, 100 mM NaCl, 0.5% sarcosyl, 0.5 mg/ml proteinase K). DNA was then precipitated with isopropanol. To screen for the full-length HTLV-1 provirus, sequence-tagged site polymerase chain reaction was carried out as described [33] using primers specified for distinct regions/genes of the HTLV-1 genome (supplementary Table S1). PCR products were resolved on 2% agarose gels.

### RNA extraction and real-time quantitative RT-PCR

Total mRNA, nuclear and cytoplasmic mRNAs from HTLV-1-infected cell clones were isolated using the PARIS kit (Ambion) according to manufacturer's instructions. Contaminating genomic DNA was removed using the turbo DNA-free kit (Ambion). Complementary DNA (cDNA) was synthesized from 500 ng of RNA in a total volume of 10 µl with iScript reverse transcription super mix (Biorad). The cDNA used for HBZ mRNA quantitation was prepared using a gene-specific antisense primer, HBZ-R2 (see supplementary Table S 2), to avoid contamination from HTLV-1 sense strand cDNA. Real-time PCR was performed using 2 µl of the cDNA as template in a 20 µl reaction, using gene-specific primers (see supplementary Table S2 for sequences) and LightCycler DNA SYBR Green I master mix (Roche applied science) in a LightCycler thermal cycler (Roche Diagnostics). The mRNA level in each sample was normalized to that of the β-actin mRNA. Relative mRNA levels were calculated using the 2^−ΔCt^ method [34]. To determine the nuclear-to-cytoplasmic ratio of a given viral mRNA species, 150 ng of nuclear or 300 ng of cytoplasmic mRNA was used for cDNA synthesis. Complementary DNA from each fraction was quantified for the level of Gag-Pol, pXIII, and β-actin mRNA transcripts respectively by PCR, the relative abundance of each viral mRNA in the nuclear or cytoplasmic compartment was determined by normalizing the level of a given viral mRNA against that of the β-actin mRNA in the same mRNA preparation. The nuclear-to-cytoplasmic ratio of viral mRNAs was calculated based on the relative abundance measurements, and then plotted.

### Transfections and luciferase assays

Cells (3×10^5^) were seeded into a 24-well plate overnight. After 16 hours, DNA transfections were performed using Fugene HD reagent (Promega). Two hundred nanograms of each reporter plasmid HTLV-1 LTR-Luc, E-selectin-Luc, or RxRE-RLuc were used in the respective luciferase reporter assay. The RxRE1-RLuc contains the HTLV-1 Rex-response element downstream of the Renilla luciferase reporter (RLuc) gene located within an intron (kindly provided by Dr. Jaqueline Dudley). The amounts of Rex or HBZ expression plasmid used range from 0 to 200 ng. All transfections were performed in duplicates. The total DNA amount (500 ng) was kept constant in all transfections using an empty vector plasmid, pcDNA3.1. Twenty nanograms per well of control luciferase plasmid pGL3-Luc (firefly) or pRL-TK (renilla) were also included in each transfection. After 48 hours, cells are harvested, and luciferase activity was measured using the Dual-Luciferase Reporter Assay System (Promega) according to the manufacturer's instructions. Transfection efficiencies were normalized using either TK-renilla or PGL3-Luc. Data are mean ± s.d. from at least three independent experiments.

### Lentivirus vector preparation and transduction

The lentivirus expressing the degradation resistant mutant of IκBα, LV-ΔN-IκBα-SV-puro, has been described earlier [10]. For delivery of the anti-HBZ short hairpin RNA (shRNA), A small hairpin RNA (shRNA) expression cassette containing the HBZ shRNA sequence [30] downstream of the mouse U6 promoter was amplified by PCR and cloned into a self-inactivating lentiviral vector, SMPU [14], engineered to contain the SV-puro (the puromycin resistance gene placed under the control of the SV40 early promoter). Lentiviral vectors were prepared as previously reported [15]. HeLa-G cells were transduced with the lentiviral vector in DMEM supplemented with 10% fetal bovine serum and selected in the same medium containing puromycin (1 µg/ml).

## Supporting Information

Figure S1 **Detection of HTLV-1 proviral sequence by PCR.** Genomic DNA was isolated from MT2 (positive control), HeLa-G (negative control), and representative HTLV-1-infected clones of each of the HeLa-G and HeLa-G/ΔN-IκBα groups (G1-3 and ΔN1-2) characterized in Fig. 1B, and subjected to sequence-tagged site polymerase chain reaction using primers spanning various regions of the entire provirus. The primers used, the regions of the viral genome covered, and the sizes of the expected PCR products are listed in supplementary Table S1.
(TIF)

Figure S2 **Reactivation of latent HTLV-1 in LIC clone 3 by Tax and Rex individually or in combination.** Cells of LIC clone 3 (3×10^5^ cells per well in a 6-well plate) were transfected for 48 hours with expression constructs for Tax (Bc12-Tax) and Rex (pRSV-Rex) either individually or in combination using Fugene reagent (Promega). Whole cell lysates were analyzed by immunoblotting using antibodies against p24, Tax, Rex, and β-actin (Actin).
(TIF)

Table S1 **Primers used for sequence-tagged-site (STS) PCR detection of various regions of HTLV-1 genome.** The regions of interest (STS), the nucleotide positions correspond to each of the upstream primer sequences in the HTLV-1 genome, the PCR product sizes are indicated.
(DOCX)

Table S2 **Nucleotide sequences of primers used for RNA quantitation by real-time qPCR are listed.**
(DOCX)
