# Supplementary figures and images for: Regulation of Human T-Lymphotropic Virus Type I Latency and Reactivation by HBZ and Rex
Source: PLoS Pathog. 2014 Apr 3;10(4):e1004040. doi: 10.1371/journal.ppat.1004040 (PMC3974842; doi:10.1371/journal.ppat.1004040)

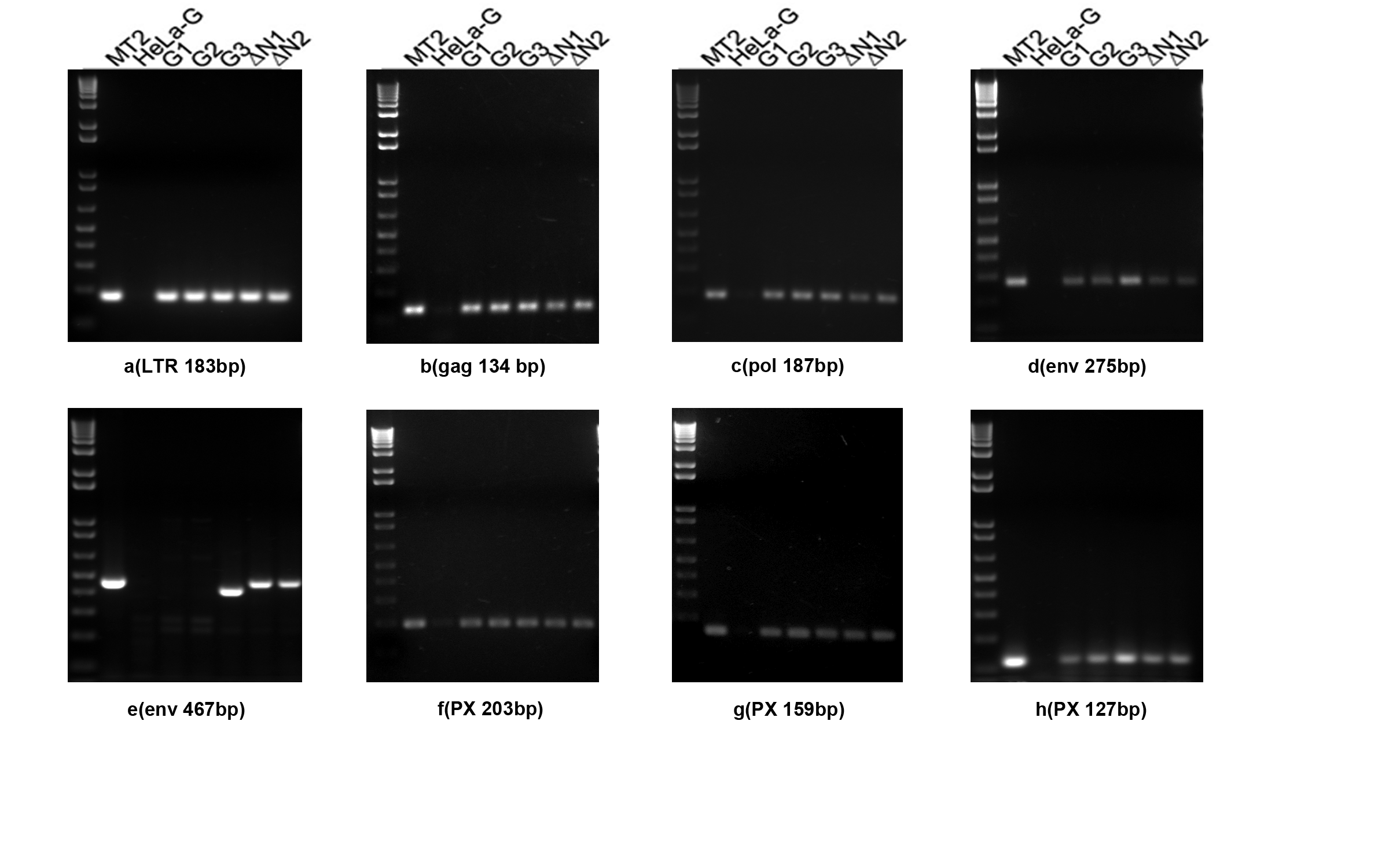

Supplement: Figure S1 — Detection of HTLV-1 proviral sequence by PCR. Genomic DNA was isolated from MT2 (positive control), HeLa-G (negative control), and representative HTLV-1-infected clones of each of the HeLa-G and HeLa-G/ΔN-IκBα groups (G1-3 and ΔN1-2) characterized in Fig. 1B, and subjected to sequence-tagged site polymerase chain reaction using primers spanning various regions of the entire provirus. The primers used, the regions of the viral genome covered, and the sizes of the expected PCR products are listed in supplementary Table S1. (TIF) [file ppat.1004040.s001.tif]

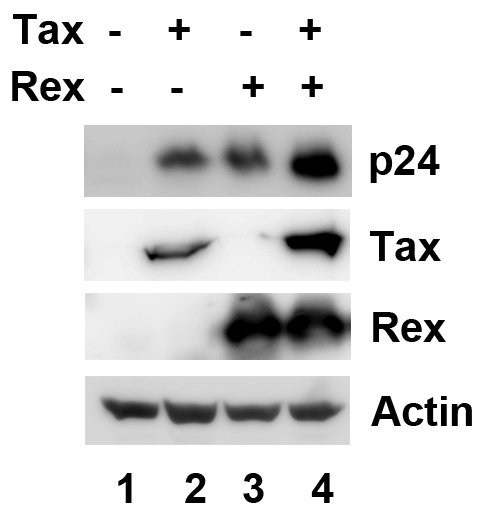

Supplement: Figure S2 — Reactivation of latent HTLV-1 in LIC clone 3 by Tax and Rex individually or in combination. Cells of LIC clone 3 (3×10^5^ cells per well in a 6-well plate) were transfected for 48 hours with expression constructs for Tax (Bc12-Tax) and Rex (pRSV-Rex) either individually or in combination using Fugene reagent (Promega). Whole cell lysates were analyzed by immunoblotting using antibodies against p24, Tax, Rex, and β-actin (Actin). (TIF) [file ppat.1004040.s002.tif]
