# Supplementary material for: Regulation of Human T-Lymphotropic Virus Type I Latency and Reactivation by HBZ and Rex
Source: PLoS Pathog. 2014 Apr 3;10(4):e1004040. doi: 10.1371/journal.ppat.1004040 (PMC3974842; doi:10.1371/journal.ppat.1004040)
Supplement: Table S1 — Primers used for sequence-tagged-site (STS) PCR detection of various regions of HTLV-1 genome. The regions of interest (STS), the nucleotide positions correspond to each of the upstream primer sequences in the HTLV-1 genome, the PCR product sizes are indicated. (DOCX) [file ppat.1004040.s003.docx]

**Supplementary Table S1: Primers used for sequence-tagged-site (STS) PCR detection of HTLV-1 genome**

| **STS** | **Primer position** | **Sequence** | **Product (bp)** |
| --- | --- | --- | --- |
| a (LTR) | 207-228 | 5-TGACCCTGCTTGCTCAACTCTA-3’  5’-CTCTCCTGGAGAGTGCTATAG-3’ | 183 |
| b (gag) | 1049-1070 | 5’- CCATCACCAGCAGCTAGATAGC-3’  5’-GCTGGTATTCTCGCCTTAATC C-3’ | 134 |
| c (pol) | 4383-4405 | 5’-CCCTACAATCCAACCAGCTCC G-3’  5’-GTGGTGAAGCTGCCATCGGGTTTT-3’ | 187 |
| d (env) | 5054-5073 | 5’- TACCATGCCACCTATTCCCT-3’  5’- GCGTCGACTAGAAGGGAGAAG-3’ | 275 |
| e (env) | 5318-5342 | 5’-CTAGTCGACGCTCCAGGATATGACC-3’  5’-CAGACCGCCACCGGTACCGCTCGGC-3’ | 467 |
| f (pXIII) | 6951-6975 | 5’-CCCACTTCCCAGGGTTTAGACAGA G-3’  5’-CTGTAGAGCTGAGCCGAT AACGCG-3’ | 203 |
| g (pXIII) | 6985-7004 | 5’- CGGATACCCAGTCTACGTGT-3’  5’- GAGCCGATAACGCGTCCATCG-3’ | 159 |
| h (pXIII) | 7223-7244 | 5’-CCAATCACTCATACAACCCCC A-3’  5’-CTGGAAAAGACAGGGTTGGGAG-3’ | 127 |
