# Supplementary material for: Regulation of Human T-Lymphotropic Virus Type I Latency and Reactivation by HBZ and Rex
Source: PLoS Pathog. 2014 Apr 3;10(4):e1004040. doi: 10.1371/journal.ppat.1004040 (PMC3974842; doi:10.1371/journal.ppat.1004040)
Supplement: Table S2 — Nucleotide sequences of primers used for RNA quantitation by real-time qPCR are listed. (DOCX) [file ppat.1004040.s004.docx]

**Supplementary Table S2: Primers used for real-time qPCR.**

| **Primer** | **Sequence** |
| --- | --- |
| HBZ-R2 | 5’-TCT TCCTCCAAGGATAATAGCCCGTCCA |
| HBZ-F | 5’- CAGTAGGGCGTGACGATGTA |
| HBZ-R | 5’- CAAGGATAATAGCCCGTCCA |
| Gag/pol-F | 5’- CCCTCCAGTTACGAT TTCCA |
| Gag/pol-R | 5’- GGCTTGGGTTTGGATGAGTA |
| Env-F | 5'- CTGTGGTGCCTCCTGAACT |
| Env-R | 5'- AAAGTGGCGAGAAACTTACCC |
| pXIII-F | 5'- ATCCCGTGGAGACTCCTCAA |
| pXIII-R | 5'- CCAAACACGTAGACTGGGTATCC |
| β-actin-F | 5'- TGAGCTGCGTGTGGCTCC |
| β-actin-R | 5'- GGCATGGGGGAGGGCATACC |
